# An AFLP-based genetic linkage map of *Plasmodium chabaudi chabaudi*

**DOI:** 10.1186/1475-2875-4-11

**Published:** 2005-02-11

**Authors:** Axel Martinelli, Paul Hunt, Richard Fawcett, Pedro VL Cravo, David Walliker, Richard Carter

**Affiliations:** 1 Institute for Immunology and Infection Research, School of Biological Science, University of Edinburgh, Ashworth Laboratories, King's Buildings, West Mains Road, Edinburgh EH9 3JT, UK; 2 Centro de Malária e Outras Doenças Tropicais/IHMT/UEI Biologia Molecular/UNL, Rua da Junqueira, 96, 1349-008, Lisbon, Portugal

## Abstract

**Background:**

*Plasmodium chabaudi chabaudi *can be considered as a rodent model of human malaria parasites in the genetic analysis of important characters such as drug resistance and immunity. Despite the availability of some genome sequence data, an extensive genetic linkage map is needed for mapping the genes involved in certain traits.

**Methods:**

The inheritance of 672 Amplified Fragment Length Polymorphism (AFLP) markers from two parental clones (AS and AJ) of *P. c. chabaudi *was determined in 28 independent recombinant progeny clones. These, AFLP markers and 42 previously mapped Restriction Fragment Length Polymorphism (RFLP) markers (used as chromosomal anchors) were organized into linkage groups using Map Manager software.

**Results:**

614 AFLP markers formed linkage groups assigned to 10 of 14 chromosomes, and 12 other linkage groups not assigned to known chromosomes. The genetic length of the genome was estimated to be about 1676 centiMorgans (cM). The mean map unit size was estimated to be 13.7 kb/cM. This was slightly less then previous estimates for the human malaria parasite, *Plasmodium falciparum*

**Conclusion:**

The *P. c. chabaudi *genetic linkage map presented here is the most extensive and highly resolved so far available for this species. It can be used in conjunction with the genome databases of *P. c chabaudi*, *P. falciparum *and *Plasmodium yoelii *to identify genes underlying important phenotypes such as drug resistance and strain-specific immunity.

## Background

*Plasmodium chabaudi chabaudi *is a malaria parasite of murine rodents. It has been widely used as a model to study various aspects of parasite biology and disease which are difficult to investigate using human malaria parasites. For instance, *P. c. chabaudi *is being used to study the genetic basis of drug resistance [1-4] and strain-specific immunity [5], because the execution and analysis of genetic crosses is relatively straightforward in this species [6]. The analysis of the genetic basis of aspects of malaria biology has been facilitated by recent developments in malaria genomics. Firstly, the *Plasmodium falciparum *genome has been fully sequenced and mapped [7] and there is also extensive sequence data now available for three of the four main malaria parasites of murine rodents [8]. Secondly, the degree of homology and conservation of gene synteny between the various species of malaria [4,9,10] allows the undertaking of comparative genomics and facilitates the elaboration of accurate genomic maps in these species.

However, a genetic linkage map of the 14 chromosomes of *P. c. chabaudi *is still important for the identification of loci which influence phenotypes such as drug resistance. A previous genetic linkage map of *P. c. chabaudi *was generated using over 40 RFLP markers [11]. However, due to the small number of markers available, this linkage map had limited usefulness. The authors have recently developed a large number of genome-wide polymorphic AFLP markers for *P. c. chabaudi *[11]. AFLP markers have previously been used to generate genetic linkage maps in another apicomplexan parasite, *Eimeria tenella *[12], as well as in *Trypanosoma brucei *[13].

This article presents a high-resolution genetic linkage map of *P. c. chabaudi *and an estimate of map unit size. The value of the genetic linkage map in the identification of genes determining selectable phenotypes is also described.

## Methods

### Mouse strains used in experiments

Inbred female CBA mice, obtained from the University of Edinburgh, were used for the growth of *P. chabaudi *parasites. Mice were housed in propylene cages with sawdust bedding and were fed on Harlan, SDS formula number I (Special Diet Services Ltd.) and drinking water supplemented with 0.05% paraminobenzoic acid (PABA) to aid parasite growth [14]. Temperature was maintained between 22 and 25°C with a 12 hour light/12 hour dark cycle.

### Parasite lines

Clones of the genetically distinct isolates AS and AJ, originally isolated from wild thicket rats, *Thamnomy rutilans *[15] were used as parents in genetic crosses.

28 recombinant clones were analysed here. 20 clones originated from a cross between AJ and AS (3CQ) (a chloroquine-resistant clone derived from AS) [1], while 8 clones originated from a cross between AJ and AS (30CQ) (a clone with higher resistance to chloroquine, derived from AS (3CQ) [16].

### Maintenance of parasites

For routine maintenance of parasites, parasitized red blood cells collected from the tail veins of infected mice were passaged in citrate saline into uninfected mice. Cryopreservation of infected blood was performed by exsanguination of mice anaesthetised with halothane. Blood was collected in a tube containing 2–3 volumes of citrate saline (0.9% NaCl, 1.5% tri sodium citrate dihydrate, adjusted to pH 7.2). The mixture was spun at 2000 rpm for five minutes, the supernatant discarded and the red cell pellet mixed with two volumes of a solution containing 28% (v/v) glycerol, 3% sorbitol and 0.65% NaCl. The mixture was then aliquoted into several glass capillaries, which were sealed by flame and deep-frozen in liquid nitrogen.

50 μl of cryopreserved blood was recovered by thawing capillaries into 10 μl of 12% NaCl and mixing for 3–5 mins. Nine volumes of 1.6% NaCl were then added dropwise and samples centrifuged at 2000 rpm for 3–5 mins. The supernatant was removed and nine volumes of 0.9% NaCl/ 0.2% dextrose solution were added dropwise. After mixing, the mixture was centrifuged again, supernatant removed and red blood cells resuspended in a 0.9% NaCl/ 0.2% dextrose solution for injection.

### Estimation of parasitaemia

Parasitaemia was estimated by microscopic observation of thin blood smears taken 4–6 days after parasite injection and stained with 20% Giemsa staining solution (BDH) for 15 minutes. Parasitaemia was estimated by calculating the percentage of red blood cells infected in at least five microscopic fields.

### Preparation of parasite DNA

Each parasite DNA preparation was obtained from five infected CBA female mice. Blood samples were taken from mice infected with AS, AJ and recombinant clones having high parasitaemias in the mid-afternoon, when parasites were trophozoites.

Host lymphocytes or nucleated cells present in the blood were removed as described previously [11]. Parasites were pelleted and stored at -70°C. DNA was extracted and purified as previously described [11] and stored at -20° for future use.

### Amplified Fragment Length Polymorphism (AFLP) technique

The AFLP method was carried out according to the original protocol [17] with slight modifications, as described by Grech *et al *[11]. Briefly, parasite genomic DNA was digested with two enzymes, *Eco*RI and *Mse*I, and ligated with adapters, to provide the complementary sequences for AFLP primers. The first round of amplification used primers containing the *Eco*RI or *Mse*I recognition sequences at their 3' end. The second round of (selective) amplification use two additional (selective) bases (3' terminus) in both primers, one of which (the *Eco*RI primer) was radiolabeled with γ-[^33^P] ATP. PCR products were run on acrylamide gels and AFLP bands visualised on autoradiography films. Polymorphic bands between the two parental strains were used as markers for the genetic linkage map.

### Organization of AFLP markers in a genetic linkage map

For every marker, the parental alleles identified in each of the progeny clones were entered in an Excel spreadsheet. The absence of a band in one parent was treated as the presence of the other parental allele at that locus. Data were then prepared for analysis with the Map Manager QTX software [18] according to the instruction manual. The dataset was designated as "Backcross" for the purpose of computer analysis.

Prior to linkage analysis, markers were tested for random assortment using a chi-square test, to exclude markers segregating in a non-random fashion from the initial analysis with Map Manager, and thus to avoid spurious linkage inferences between such markers. Because of the large number of tests for non -random assortment performed (n = 672), some markers showing apparent non-random assortment may have been falsely excluded from our analysis; i.e. some valid markers may indeed show non-random assortment and should be included. A Bonferroni correction was therefore applied to the chi-square test to decrease the stringency of the statistical test. The value of the Bonferroni correction represents the number of 'independent' comparisons and here was arbitrarily set at 24. This value was chosen as representing the likely number of chromosomal fragments at meiosis, and is supported by data presented in this paper. Markers within these fragments are not independently inherited. The chosen value (24) is a compromise between 672 (which assumes that all 672 markers are independently inherited) and 14 (the number of chromosomes, and which assumes that no pair of markers on one chromosome are inherited independently). Markers not following random assortment in the initial test were thus divided into two groups, i.e. those segregating in a non-random fashion before and after Bonferroni correction, and those segregating in a non-random fashion before but not after the Bonferroni correction. The markers in the latter group were added separately after linkage groups had been determined (see below).

Linkage groups using AFLP and RFLP markers were formed with an initial p-value of 0.0001 using the "Make Linkage Groups" command in Map Manager. p-values in Map Manager indicate the probability of a Type 1 error; that is, the probability of a false positive linkage. Following formation of linkage groups, the p-value was raised to 0.001. Linkage at p < 0.001 is considered significant. Using the command "Distribute", linkage groups were brought together. Then, other previously unlinked markers were allocated to these new linkage groups, again using the "Distribute" command. Markers with non-random assortment after statistical analysis without Bonferroni correction were added next, and those still segregating in a non-random fashion after Bonferroni correction were added last. The "Ripple" function was then used to position markers in an order which maximizes the total LOD (logarithmic odds) score for linkage. The software also estimated the optimum order and genetic distance between markers in centiMorgans (cM) by using the "Kosambi" function in the software. It was then possible to calculate a map unit size (i.e the physical distance corresponding to 1 cM).

The presence of 42 previously characterised RFLP markers which had been physically mapped onto *P. c. chabaudi *chromosomes [1] served as anchors for the placement of AFLP linkage groups onto specific chromosomes.

## Results

The inheritance of 672 AFLP markers was determined in 28 progeny clones derived from two crosses between *P. c. chabaudi *AJ and either clone AS (3CQ) or AS (30CQ). The majority of the AFLP markers showed independent assortment in the 28 progeny clones, as illustrated previously [11]. However, 66 markers failed the chi-square test at 5%, 15 of which failed it after the Bonferroni correction. Markers were allocated to linkage groups using the Map Manager program and groups assigned to chromosomes using 42 previously mapped RFLP markers as anchors [1]. Estimated numbers of recombination events, genetic lengths of chromosomes and recombination frequencies were also determined for the identified chromosomes using Map Manager.

### Allocation of markers to linkage groups

The 672 AFLP markers formed a total of 22 linkage groups with a final p-value of 0.001. Additional file 1 summarises the numbers of AFLP and RFLP markers assigned to each chromosome or to unassigned linkage groups, the estimated physical size of each chromosome [19] and the number of AFLP markers per Mb. 400 AFLP markers in 10 linkage groups could be assigned to *P. c. chabaudi *chromosomes 1 and 5–13, by virtue of their linkage to RFLP markers previously assigned to specific chromosomes by physical mapping [1]. 272 AFLP markers could not be assigned to a specific chromosome. 214 were placed in 12 unassigned linkage groups, each with between 2 and 51 AFLP markers. At least four of these linkage groups are likely to map to chromosomes 2, 3, 4 or 14. The failure to assign these linkage groups occurred because RFLP markers previously mapping to chromosomes 2, 3, 4 and 14 were not allocated to linkage groups. This was probably due to insufficient characterization of the inheritance patterns of these RFLP anchors which were determined in a small number of recombinant clones [1]. For instance, the inheritance of a RFLP marker assigned to chromosome 2, Ca-ATPase, was only determined for 7 out of the 28 recombinant clones. No independent physical mapping of unassigned linkage groups was attempted here.

58 AFLP markers, 21 of which segregated in a non-random fashion, could not be allocated to any linkage groups. Physical mapping of these markers would be required to assign them to specific chromosomes. Alternatively, unassigned linkage groups or unallocated markers might map to the small mitochondrial or apicoplast genomes, although these combined represent only 0.2% of the genome. Several RFLP markers could not be allocated to linkage groups by the Map Manager software, probably due to the small numbers of clones analysed for these markers. These markers were added to assigned linkage groups according to their chromosomal assignment, as previously determined by physical mapping [1].

With the exception of chromosomes 2, 3, 4 and 14 (discussed above), chromosomes 9 and 10 showed the lowest density per Mb of AFLP markers. Chromosome 7 showed the highest density. This may simply reflect natural variation in the frequency of AFLP polymorphisms on particular chromosomes. However, for chromosomes with a low apparent density of AFLP markers such as chromosomes 9 and 10, it is likely that some of the markers in unassigned linkage groups would physically map to these chromosomes. These unassigned groups may not show genetic linkage with (groups of) *assigned *markers because of factors such as a high rate of recombination *between *two linkage groups (one linked to the RFLP anchor) or because of an intervening region with a low density of AFLP markers. Both factors, or a combination of the two, may prevent two physically linked groups from being identified as genetically linked. Conversely an apparent unusually high frequency of AFLP markers (as in chromosome 7) may arise from strong linkage disequilibrium between loci on two different chromosomes. Some markers located on one chromosome may thus appear to be genetically linked to markers on another. This could arise where one locus exerts a strong constraint on another unlinked locus. For instance, the AJ allele of an enzyme in a metabolic pathway may only function with the presence of the product of the AJ allele encoding another enzyme in the same pathway. This constraint might be structural or functional. The same may be true of AS alleles of the same enzymes. In this case, the genes encoding these enzymes, and markers strongly linked to them, may appear in the same genetic linkage group.

### Order of markers in the linkage groups

AFLP markers were initially ordered on the linkage groups as described in Materials and Methods. After inspection of the predicted marker order, occasional manual adjustments were made to correct markers which appeared to be inappropriately positioned.

**Figure 1:**
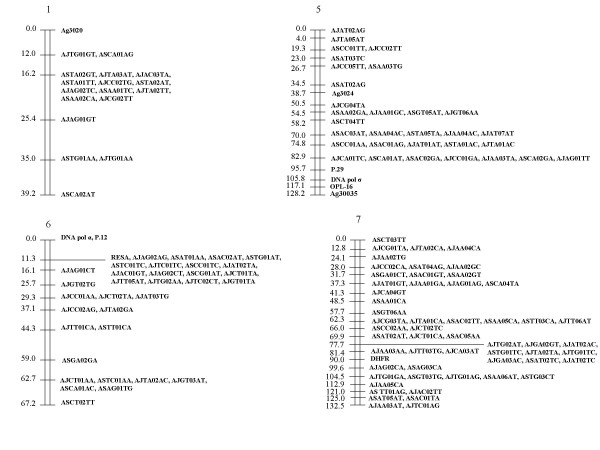
**Linkage map for chromosomes 1, 5, 6 and 7 of the *P. c. chabaudi *genome. **The AFLP and RFLP markers assigned to chromosomes are displayed with genetic distances (in cM). AFLP markers were named as follows: the first two letters identify the clone to which a marker is specific, the next two letters indicate the *Eco*RI primer selective bases, the numbers identify the marker for that clone and primer combination in order of its molecular size, and the last two letters identify the *Mse*I selective bases. RFLP markers were based on genes previously identified [1].

**Figure 2:**
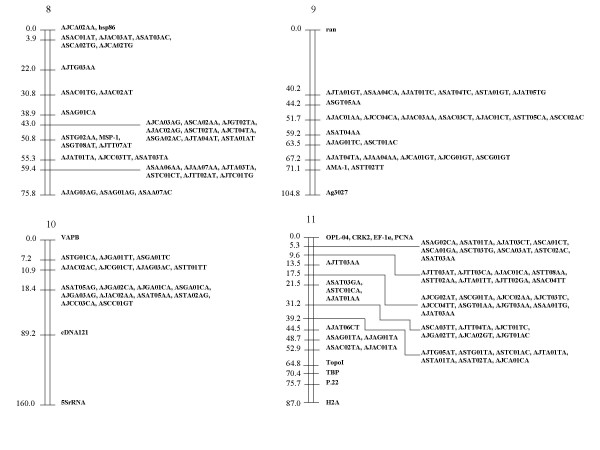
**Linkage map for chromosomes 8–11 of the *P. c. chabaudi *genome. **The AFLP and RFLP markers assigned to chromosomes are displayed with genetic distances (in cM).

Because Map Manager failed to allocate some RFLP markers to an assigned linkage group, these were positioned manually. The final distribution of the markers on the 10 linkage groups assigned to chromosomes is shown in Fig. 1, 2, 3 (See also Additional file 2, Additional file 3.  and Additional file 4). 7 unassigned linkage groups containing 9 or more markers each are shown in Fig. 4 (see also Additional file 5).

**Figure 3:**
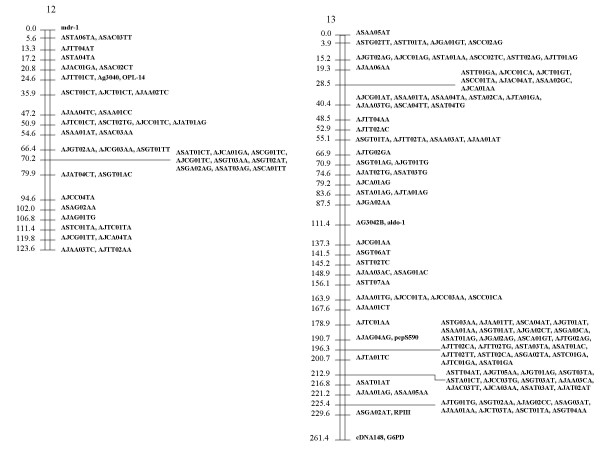
**Linkage map for chromosomes 12 and 13 of the *P. c. chabaudi *genome. **The AFLP and RFLP markers assigned to chromosomes are displayed with genetic distances (in cM).

**Figure 4:**
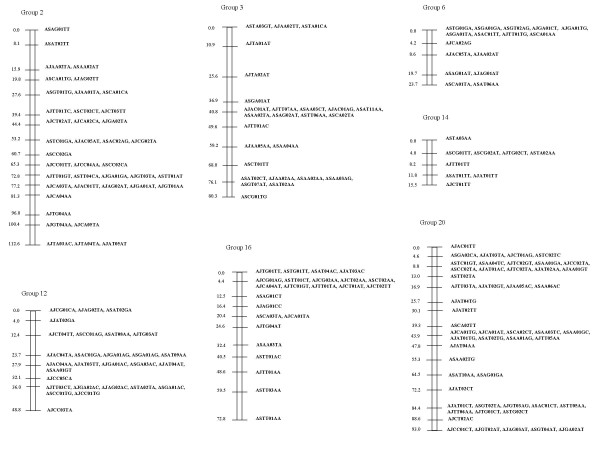
**Unassigned linkage groups containing more than 8 markers. **The AFLP markers assigned to unassigned linkage groups are displayed with genetic distances (in cM)

### Number of recombination events per chromosome

If markers and recombination events were both uniformly distributed across the genome, then we would expect the number of predicted recombination events (totalled from 28 clones characterised here) in each chromosome to correlate with its physical size. The predicted total number of recombination events occuring in each linkage group is shown in the Additional file 1. Uniformity was evaluated by comparison of the frequency of recombination events (from the 28 clones) in each chromosome. This varies between 6.7/Mb (chromosome 10)) and 31.1/Mb (chromosome 7), with an overall value of 13.3/Mb. Chromosomes 1 and 11 also showed low frequencies. These differences may reflect natural variation in recombination rates across the genome. However, other factors may also contribute. For instance, there is likely to be a systematic underestimation of recombination frequency because the physical extent of the linkage groups assigned to particular chromosomes will be less than their actual size. For instance, if the chromosome 10 linkage group extends across only half of the chromosome, then the real density of markers and recombination events (per Mb) is likely to be about twice the apparent value given. Indeed, Additional file 1 shows that when data from the unassigned linkage groups are included, the recombination frequency (across the whole genome) increases to 15.9 events per Mb. Regardless of the number of markers assigned to each chromosome, we would expect the number of recombination events per AFLP marker to remain relatively constant, if both the frequency of polymorphism and the rate of recombination vary little between chromosomes. This is indeed the case. For the different chromosomes, the measure varies only between 0.4 and 0.9 recombination events per AFLP marker (see Additional file 1).

Assuming that the *P. c. chabaudi *genome consists of about 20 Mb [19] the value of 13.3 recombination events in 28 recombinant clones/Mb converts to about 9.5 recombination events/clone/genome which is very close to the value of 10 estimated for *Plasmodium falciparum *[21].

A significant number of double crossover events around a single AFLP marker were observed in many linkage groups. Any such occurrences were re-evaluated on the original X-ray film to detect any possible errors or ambiguous bands. Many distinct double crossover events were observed. The same phenomenon was also commonly observed in *P. falciparum*, and were interpreted as being due to non-reciprocal conversion events [20].

### Genetic length of linkage groups

**Figure 5:**
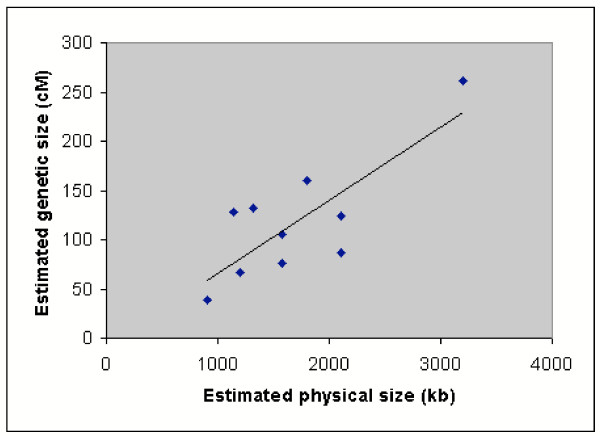
**Relationship between genetic size and physical size of each chromosome. **The relationship between the estimated genetic sizes and physical sizes of chromosomes 1, 5–13 (Table 1) is shown. The correlation between these two variables is 0.794.

The apparent genetic length of each linkage group in cM was calculated by Map Manager based on the number of recombination events (Additional file 1). When added together, the linkage groups assigned to chromosomes combined to give a total genetic length of 1180 cM and the unassigned linkage groups a further 497 cM, giving a total for the genome of 1676 cM. Due to the limited number of clones available (28), the smallest genetic distance that could be measured between two markers was approximately 3.6 cM, corresponding to the presence of a single recombination event between two markers in 28 clones. In general, the estimated genetic lengths of the linkage groups assigned to chromosomes 1, 5–13 increased with the estimated physical sizes of the chromosomes (Figure 5). The Pearson correlation coefficient was 0.794 (p < 0.005). The estimated sizes of map unit for chromosomes 1 and 5–13 are shown in the Additional file 1. These values varied from 8.9 kb/cM (chromosome 5) to 24.1 kb/cM (chromosome 11) with an overall estimated mean of 15.1 kb/cM. It is likely that there is some overestimation of physical size of a map unit because individual identified linkage groups are unlikely to cover the full extent of any chromosome. Indeed, when the genetic lengths of the unassigned linkage groups are included in the analysis, the overall map unit size is reduced to 13.7 kb/cM. The inclusion of additional unallocated markers may reduce this value even further.

However, some overestimation of genetic length in linkage groups is also possible, which leads to an underestimation of map unit size. For instance, Figure 2 shows that chromosomes 9 and 10 both show two abnormally large sections bounded by RFLP anchors without intervening AFLP markers. Specifically, chromosome 9 shows, at one end, an RFLP marker, *ran*, 40 cM from its nearest AFLP marker. At its other end, an RFLP marker, Ag 3027, lies about 35 cM from its nearest AFLP marker. Chromosome 10 has RFLP marker cDNA121 about 70 cM from its nearest AFLP marker and RFLP marker, 5S rRNA, a further 70 cM distant. These large gaps may be artefacts which arise for two reasons. Firstly, some unreliability in the typing of clones using RFLPs was previously noticed [4]. Secondly, the inheritance patterns of these markers were not determined in all 28 recombinant clones. Markers *ran*, Ag3027, cDNA121 and 5S rRNA were typed for only 9, 17, 9 and 16 clones, respectively. The characterisation of inheritance of RFLP markers in all of the 28 progeny clones, and the correction of possible mistakes may reduce the estimated genetic length. This would lead to an increase in map unit size. Nevertheless, it is notable that the value reported above (13.7 kb/cM) is close to estimates made for *P. falciparum *(15–30 kb/cM [21] or 17 kb/cM [20]), although slightly smaller. It is likely that the recombination rate may vary within as well as between chromosomes or genomic loci [22].

### Estimate of potential alleles due to indel mutations

Of the 400 AFLP markers placed on chromosomes, 37 AS-AJ pairs (74 markers i.e. 18% of the total) shared the same selective bases at both primer ends and showed complementary segregation in the cross-progeny clones. Most of these markers also differed in size by only a few base pairs. They are likely to be alleles at the same loci. This was confirmed by sequencing two such pairs, namely ASTA01AC and AJTA01AC (chromosome 5), and ASTT02CA and AJTT02CA (chromosome 13) (sequence data not shown). This suggests that a significant proportion of the polymorphisms observed between AS and AJ may be due to small insertions or deletions. In fact, it was observed that small indels tend to occur in introns or intergenic regions (data not shown).

### Reliability of the AFLP markers in the progeny clones

A few AFLP markers which were originally identified between clones AJ and AS [11] were not found in the progeny clones. Also, a few bands appeared in the progeny clones that were absent in the parents. All of these markers were ignored during the generation of the linkage map. It is possible that such markers could indicate genetic re-arrangements in the drug-resistant clones AS (3CQ) and AS (30CQ). However, they did not segregate with chloroquine resistance phenotype (data not shown). Some other markers were difficult to investigate because of their proximity to other bands or their location at the bottom of the gel, where bands tend to be fuzzier and more difficult to interpret.

### Effect of typing mistakes in the markers

Ongoing work on linkage between chloroquine resistance and markers on chromosome 11 [4] suggested that AFLP and/or RFLP markers were occasionally incorrectly characterised in one ore more of the 28 clones. To test the effect of incorrect typing in AFLP markers, some deliberate mistakes were introduced by changing the parent from which a particular marker was inherited in a particular recombinant clone. The effect of such changes ranged from the appearance or disappearance of predicted double-crossover events and consequent change in the estimated genetic length, to a larger scale change in the order of markers within a linkage group. Occasionally markers were reallocated to a different linkage group. It was concluded that patterns of linkage may be sensitive to errors in genotyping individual clones.

## Discussion

Genetic or physical linkage maps have been determined and reported for a number of apicomplexan parasites, including *P. falciparum *[20], *P. c. chabaudi *[11], *Eimeria tenella *[13], *Toxoplasma gondii *[23], *Theileria parva *[24] and *Cryptosporidium parvum *[25]. The map reported here is very extensive in terms of the numbers and density of markers included. Only the genetic map of *P. falciparum *exceeds its resolution

Of the AFLP markers analysed, most were assigned to 10 of the 14 chromosomes, while some were placed on 12 unassigned linkage groups, which probably include groups located on chromosomes 2, 3, 4 and 14. The remaining AFLP markers could not be allocated to any linkage group. Unallocated AFLP markers and unassigned linkage groups may arise in a number of possible ways, discussed in the Results section above, including mistakes or considerable gaps in the recorded inheritance pattern of RFLP markers and, to a lesser extent, AFLP markers. Other factors include variations in the density of AFLP markers or polymorphism in general, areas of the genome where the rate of recombination is particularly high, linkage disequilibrium between loci on different chromosomes and non-random representation of clones in our sample. The numbers of markers allocated, the approximate genetic lengths and the number of recombination events were estimated for each of the linkage groups. The genetic length of the entire genome was estimated to be 1684 cM and the overall size of map unit 13.7 kb/cM. The genetic length and number of recombination events were expected to increase with the size of chromosomes. This was generally found to be the case, although a number of factors may influence these data. These factors include the failure to assign some linkage groups, incomplete or incorrect inheritance data, particularly for RFLPs, variation in the frequency of AFLP markers across the genome, variation in the recombination rate across the genome, and incorrect assignation of some linkage groups due to linkage disequilibrium.

The presence and frequency of small indel mutations was confirmed. These markers could prove suitable for rapid typing of clones by size polymorphism and quantitative analysis by Real Time Quantitative PCR.

The generation of a complete AFLP genetic linkage map for *P. chabaudi *was originally conceived as an essential step towards the identification of loci linked to genes encoding important phenotypes, such as drug resistance. Indeed, the identification of a locus underlying chloroquine resistance in *P. chabaudi *within approximately 250 kb of chromosome 11 [4] relied upon elements of the present map in the analysis of linkage between phenotype (chloroquine resistance) and genotype (inheritance of parental AFLP markers) in individual recombinant clones. However we have also developed a novel strategy called Linkage Group Selection [5,26] which more rapidly identifies loci linked to genes underlying selectable phenotypes, such as drug resistance. For example, a drug resistant parasite is crossed with a genetically different drug sensitive parasite. The uncloned recombinant progeny are drug treated, and AFLP markers which are linked to loci underlying drug resistance may be identified as those reduced in their representation or intensity [27]. A genetic linkage map enables us to determine whether AFLP markers which are significantly reduced in intensity lie in the same linkage group, prior to further sequence analysis. Genome sequence data are now available for *P. c. chabaudi *(partial) [8] and *P. falciparum *(complete) [7], and sequenced AFLP markers can sometimes be mapped to the *P. falciparum *genome. Because of the extensive gene synteny between *P. chabaudi *(and other rodent malarias) and *P. falciparum *[4,9,10], markers closely linked in *P. falciparum *are likely to be closely linked in *P. chabaudi *too.

The correspondence between the genetic linkage map reported here and the mapping of AFLP markers to the *P. falciparum *genome in the studies discussed above [4,5,26] has increased our confidence both in the genetic linkage map reported here, and the extent of gene synteny between the *P. c. chabaudi*, *Plsmodium yoelii *and *P. falciparum *genomes [9,10]. The existence of a rodent malaria genome map, complete with syntenic relationships between it and the *P. falciparum *genome (Taco Kooij and Andy Waters, personal communication), will allow us to assign unallocated AFLP markers and unassigned linkage groups to particular chromosomes on the assumption that gene synteny is conserved.

## Authors' Contributions

AM characterized the AFLP markers in the cross progeny, generated the genetic linkage map and drafted the article, PH helped in the generation of the linkage map, analysed genetic data from it and drafted the article, RF helped in the characterization of the AFLP markers in the cross progeny, PC and DW provided the recombinant clones and revised the article, RC designed and coordinated the study, revised the article and gave final approval. All authors read and approved the final manuscript.

## Supplementary Material

Additional File 1 The file (.XLS) contains the following data for markers assigned to chromosomes and, where possible, for markers in unassigned linkage groups and the overall genome: – physical size of each chromosome (Mb), the number of markers (either RFLP or AFLP and total), the number of AFLP markers per Mb, the number of recombination events predicted for the 28 clones, the frequency of recombination events per Mb and per AFLP marker, the predicted genetic length of all linkage groups, and the estimated size of map unit (kb/cM). Click here for file

Additional File 2 This file is the original PPT files from which figure 1 was derived.Figures 1-3 contain the linkage map for the chromosomes 1 and 5-13. Click here for file

Additional File 3 This file is the original PPT files from which figure 2 was derived.Figures 1-3 contain the linkage map for the chromosomes 1 and 5-13. Click here for file

Additional File 4 This file is the original PPT files from which figure 3 was derived.Figures 1-3 contain the linkage map for the chromosomes 1 and 5-13. Click here for file

Additional File 5 This file is the original PPT files from which figure 4 was derived. Figure 4 contains various unassigned linkage groups. Click here for file
