# Supplementary material for: An AFLP-based genetic linkage map of Plasmodium chabaudi chabaudi
Source: Malar J. 2005 Feb 11;4:11. doi: 10.1186/1475-2875-4-11 (PMC550669; doi:10.1186/1475-2875-4-11)
Supplement: Additional File 2 — This file is the original PPT files from which figure 1 was derived.Figures 1-3 contain the linkage map for the chromosomes 1 and 5-13. [file 1475-2875-4-11-S2.ppt]

## Slide 1
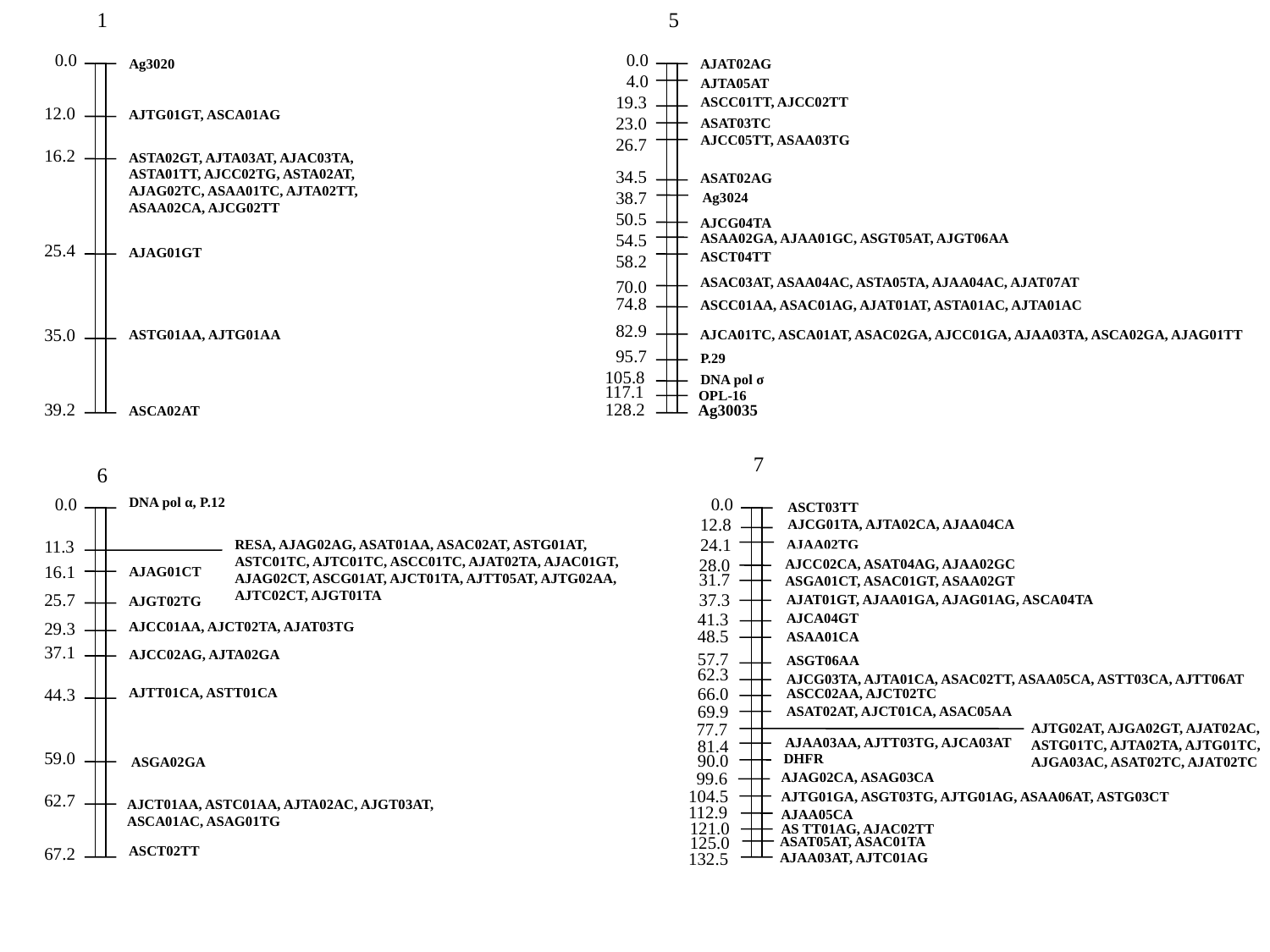

1
5
0.0
0.0
Ag3020
AJAT02AG
4.0
AJTA05AT
19.3
ASCC01TT, AJCC02TT
12.0
AJTG01GT, ASCA01AG
23.0
ASAT03TC
AJCC05TT, ASAA03TG
26.7
16.2
ASTA02GT, AJTA03AT, AJAC03TA,
ASTA01TT, AJCC02TG, ASTA02AT,
AJAG02TC, ASAA01TC, AJTA02TT,
ASAA02CA, AJCG02TT
34.5
ASAT02AG
38.7
Ag3024
50.5
AJCG04TA
54.5
ASAA02GA, AJAA01GC, ASGT05AT, AJGT06AA
25.4
AJAG01GT
ASCT04TT
58.2
ASAC03AT, ASAA04AC, ASTA05TA, AJAA04AC, AJAT07AT
70.0
74.8
ASCC01AA, ASAC01AG, AJAT01AT, ASTA01AC, AJTA01AC
82.9
35.0
ASTG01AA, AJTG01AA
AJCA01TC, ASCA01AT, ASAC02GA, AJCC01GA, AJAA03TA, ASCA02GA, AJAG01TT
95.7
P.29
105.8
DNA pol σ
117.1
OPL-16
Ag30035
39.2
128.2
ASCA02AT
7
6
0.0
DNA pol α, P.12
0.0
ASCT03TT
12.8
AJCG01TA, AJTA02CA, AJAA04CA
24.1
11.3
RESA, AJAG02AG, ASAT01AA, ASAC02AT, ASTG01AT, ASTC01TC, AJTC01TC, ASCC01TC, AJAT02TA, AJAC01GT, AJAG02CT, ASCG01AT, AJCT01TA, AJTT05AT, AJTG02AA, AJTC02CT, AJGT01TA
AJAA02TG
28.0
AJCC02CA, ASAT04AG, AJAA02GC
16.1
AJAG01CT
31.7
ASGA01CT, ASAC01GT, ASAA02GT
25.7
37.3
AJAT01GT, AJAA01GA, AJAG01AG, ASCA04TA
AJGT02TG
41.3
AJCA04GT
29.3
AJCC01AA, AJCT02TA, AJAT03TG
48.5
ASAA01CA
37.1
AJCC02AG, AJTA02GA
57.7
ASGT06AA
62.3
AJCG03TA, AJTA01CA, ASAC02TT, ASAA05CA, ASTT03CA, AJTT06AT
66.0
44.3
AJTT01CA, ASTT01CA
ASCC02AA, AJCT02TC
69.9
ASAT02AT, AJCT01CA, ASAC05AA
77.7
AJTG02AT, AJGA02GT, AJAT02AC,
ASTG01TC, AJTA02TA, AJTG01TC,
AJGA03AC, ASAT02TC, AJAT02TC
AJAA03AA, AJTT03TG, AJCA03AT
81.4
59.0
90.0
DHFR
ASGA02GA
99.6
AJAG02CA, ASAG03CA
104.5
AJTG01GA, ASGT03TG, AJTG01AG, ASAA06AT, ASTG03CT
62.7
AJCT01AA, ASTC01AA, AJTA02AC, AJGT03AT,
ASCA01AC, ASAG01TG
112.9
AJAA05CA
121.0
AS TT01AG, AJAC02TT
125.0
ASAT05AT, ASAC01TA
67.2
ASCT02TT
132.5
AJAA03AT, AJTC01AG
