# Supplementary material for: An AFLP-based genetic linkage map of Plasmodium chabaudi chabaudi
Source: Malar J. 2005 Feb 11;4:11. doi: 10.1186/1475-2875-4-11 (PMC550669; doi:10.1186/1475-2875-4-11)
Supplement: Additional File 3 — This file is the original PPT files from which figure 2 was derived.Figures 1-3 contain the linkage map for the chromosomes 1 and 5-13. [file 1475-2875-4-11-S3.ppt]

## Slide 1
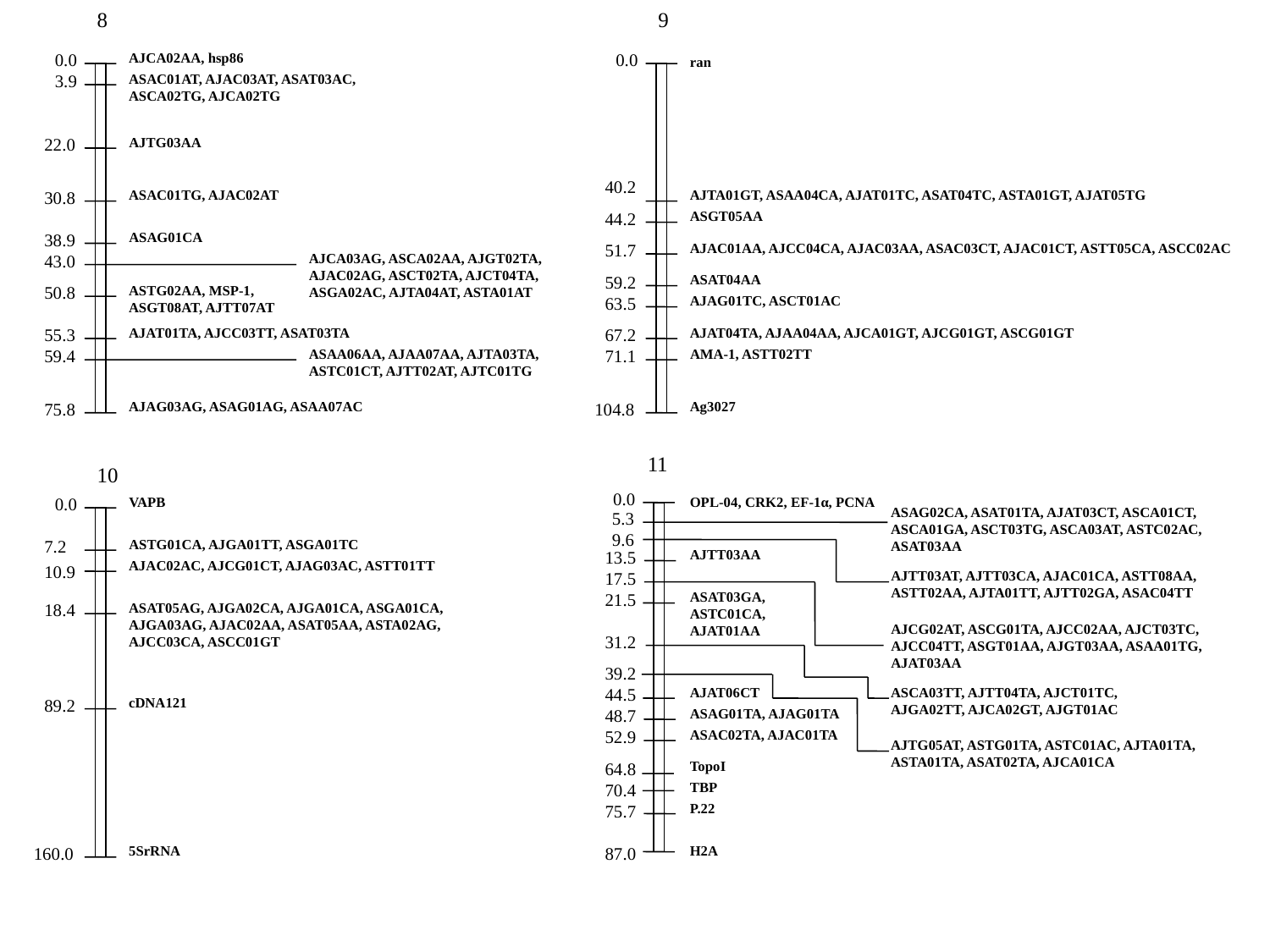

8
9
0.0
AJCA02AA, hsp86
0.0
ran
3.9
ASAC01AT, AJAC03AT, ASAT03AC, ASCA02TG, AJCA02TG
22.0
AJTG03AA
40.2
30.8
ASAC01TG, AJAC02AT
AJTA01GT, ASAA04CA, AJAT01TC, ASAT04TC, ASTA01GT, AJAT05TG
44.2
ASGT05AA
38.9
ASAG01CA
51.7
AJAC01AA, AJCC04CA, AJAC03AA, ASAC03CT, AJAC01CT, ASTT05CA, ASCC02AC
43.0
AJCA03AG, ASCA02AA, AJGT02TA, AJAC02AG, ASCT02TA, AJCT04TA, ASGA02AC, AJTA04AT, ASTA01AT
59.2
ASAT04AA
50.8
ASTG02AA, MSP-1,
ASGT08AT, AJTT07AT
63.5
AJAG01TC, ASCT01AC
55.3
AJAT01TA, AJCC03TT, ASAT03TA
67.2
AJAT04TA, AJAA04AA, AJCA01GT, AJCG01GT, ASCG01GT
59.4
ASAA06AA, AJAA07AA, AJTA03TA,
ASTC01CT, AJTT02AT, AJTC01TG
71.1
AMA-1, ASTT02TT
75.8
AJAG03AG, ASAG01AG, ASAA07AC
104.8
Ag3027
11
10
0.0
0.0
VAPB
OPL-04, CRK2, EF-1α, PCNA
ASAG02CA, ASAT01TA, AJAT03CT, ASCA01CT, ASCA01GA, ASCT03TG, ASCA03AT, ASTC02AC, ASAT03AA
5.3
9.6
7.2
ASTG01CA, AJGA01TT, ASGA01TC
13.5
AJTT03AA
AJAC02AC, AJCG01CT, AJAG03AC, ASTT01TT
10.9
17.5
AJTT03AT, AJTT03CA, AJAC01CA, ASTT08AA, ASTT02AA, AJTA01TT, AJTT02GA, ASAC04TT
21.5
ASAT03GA, ASTC01CA, AJAT01AA
18.4
ASAT05AG, AJGA02CA, AJGA01CA, ASGA01CA, AJGA03AG, AJAC02AA, ASAT05AA, ASTA02AG, AJCC03CA, ASCC01GT
AJCG02AT, ASCG01TA, AJCC02AA, AJCT03TC, AJCC04TT, ASGT01AA, AJGT03AA, ASAA01TG, AJAT03AA
31.2
39.2
44.5
AJAT06CT
ASCA03TT, AJTT04TA, AJCT01TC, AJGA02TT, AJCA02GT, AJGT01AC
89.2
cDNA121
48.7
ASAG01TA, AJAG01TA
52.9
ASAC02TA, AJAC01TA
AJTG05AT, ASTG01TA, ASTC01AC, AJTA01TA, ASTA01TA, ASAT02TA, AJCA01CA
64.8
TopoI
70.4
TBP
75.7
P.22
160.0
5SrRNA
87.0
H2A
