# Supplementary material for: An AFLP-based genetic linkage map of Plasmodium chabaudi chabaudi
Source: Malar J. 2005 Feb 11;4:11. doi: 10.1186/1475-2875-4-11 (PMC550669; doi:10.1186/1475-2875-4-11)
Supplement: Additional File 4 — This file is the original PPT files from which figure 3 was derived.Figures 1-3 contain the linkage map for the chromosomes 1 and 5-13. [file 1475-2875-4-11-S4.ppt]

## Slide 1
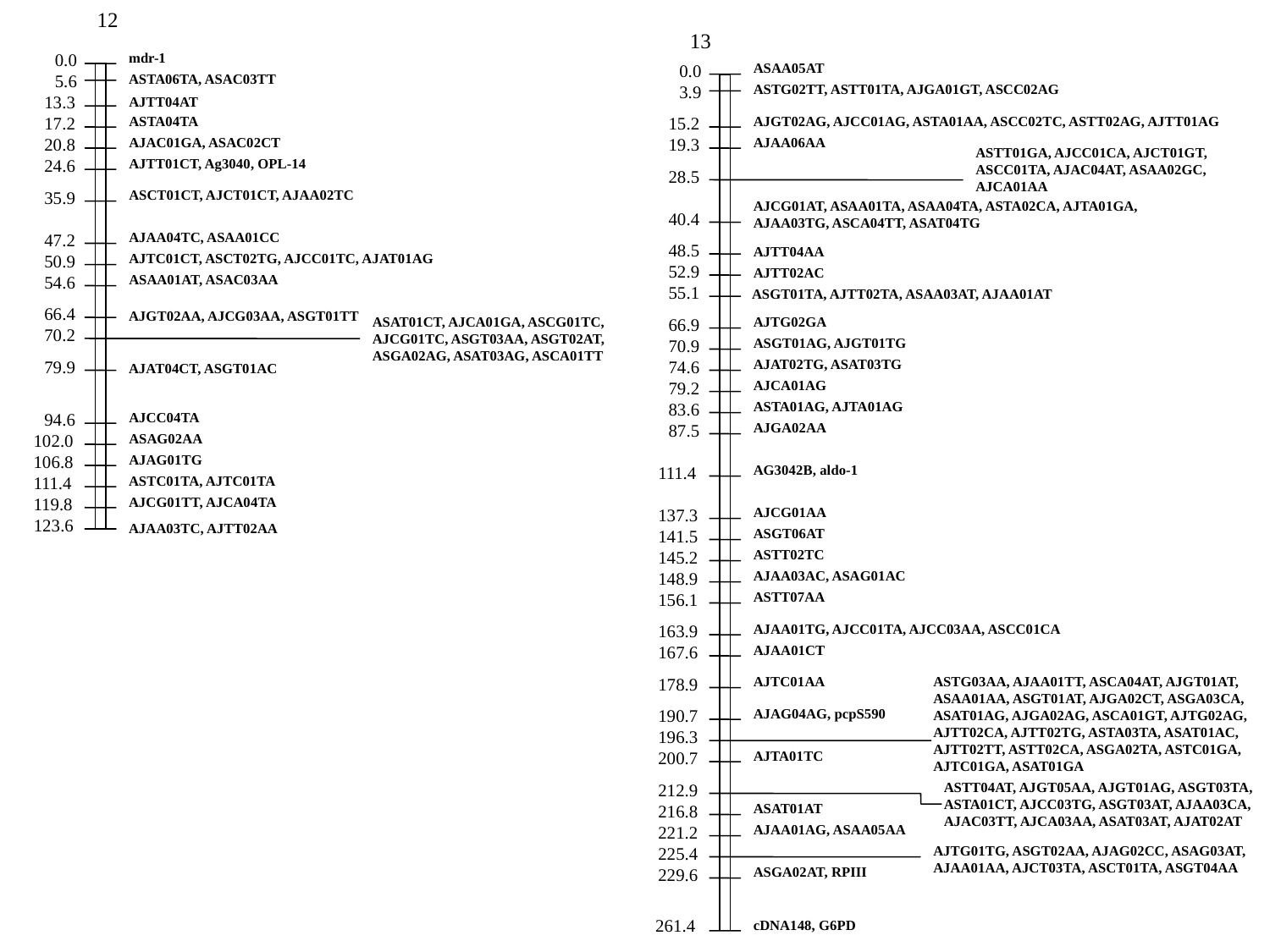

12
13
0.0
mdr-1
0.0
ASAA05AT
5.6
ASTA06TA, ASAC03TT
3.9
ASTG02TT, ASTT01TA, AJGA01GT, ASCC02AG
13.3
AJTT04AT
17.2
ASTA04TA
15.2
AJGT02AG, AJCC01AG, ASTA01AA, ASCC02TC, ASTT02AG, AJTT01AG
20.8
AJAC01GA, ASAC02CT
19.3
AJAA06AA
ASTT01GA, AJCC01CA, AJCT01GT, ASCC01TA, AJAC04AT, ASAA02GC, AJCA01AA
24.6
AJTT01CT, Ag3040, OPL-14
28.5
35.9
ASCT01CT, AJCT01CT, AJAA02TC
AJCG01AT, ASAA01TA, ASAA04TA, ASTA02CA, AJTA01GA, AJAA03TG, ASCA04TT, ASAT04TG
40.4
47.2
AJAA04TC, ASAA01CC
48.5
AJTT04AA
50.9
AJTC01CT, ASCT02TG, AJCC01TC, AJAT01AG
52.9
AJTT02AC
54.6
ASAA01AT, ASAC03AA
55.1
ASGT01TA, AJTT02TA, ASAA03AT, AJAA01AT
66.4
AJGT02AA, AJCG03AA, ASGT01TT
ASAT01CT, AJCA01GA, ASCG01TC, AJCG01TC, ASGT03AA, ASGT02AT, ASGA02AG, ASAT03AG, ASCA01TT
66.9
AJTG02GA
70.2
70.9
ASGT01AG, AJGT01TG
79.9
74.6
AJAT02TG, ASAT03TG
AJAT04CT, ASGT01AC
79.2
AJCA01AG
83.6
ASTA01AG, AJTA01AG
94.6
AJCC04TA
87.5
AJGA02AA
102.0
ASAG02AA
106.8
AJAG01TG
111.4
AG3042B, aldo-1
111.4
ASTC01TA, AJTC01TA
119.8
AJCG01TT, AJCA04TA
137.3
AJCG01AA
123.6
AJAA03TC, AJTT02AA
141.5
ASGT06AT
145.2
ASTT02TC
148.9
AJAA03AC, ASAG01AC
156.1
ASTT07AA
163.9
AJAA01TG, AJCC01TA, AJCC03AA, ASCC01CA
167.6
AJAA01CT
178.9
AJTC01AA
ASTG03AA, AJAA01TT, ASCA04AT, AJGT01AT, ASAA01AA, ASGT01AT, AJGA02CT, ASGA03CA, ASAT01AG, AJGA02AG, ASCA01GT, AJTG02AG, AJTT02CA, AJTT02TG, ASTA03TA, ASAT01AC, AJTT02TT, ASTT02CA, ASGA02TA, ASTC01GA, AJTC01GA, ASAT01GA
190.7
AJAG04AG, pcpS590
196.3
200.7
AJTA01TC
212.9
ASTT04AT, AJGT05AA, AJGT01AG, ASGT03TA, ASTA01CT, AJCC03TG, ASGT03AT, AJAA03CA, AJAC03TT, AJCA03AA, ASAT03AT, AJAT02AT
216.8
ASAT01AT
221.2
AJAA01AG, ASAA05AA
225.4
AJTG01TG, ASGT02AA, AJAG02CC, ASAG03AT, AJAA01AA, AJCT03TA, ASCT01TA, ASGT04AA
229.6
ASGA02AT, RPIII
261.4
cDNA148, G6PD
