# Supplementary material for: An AFLP-based genetic linkage map of Plasmodium chabaudi chabaudi
Source: Malar J. 2005 Feb 11;4:11. doi: 10.1186/1475-2875-4-11 (PMC550669; doi:10.1186/1475-2875-4-11)
Supplement: Additional File 5 — This file is the original PPT files from which figure 4 was derived. Figure 4 contains various unassigned linkage groups. [file 1475-2875-4-11-S5.ppt]

## Slide 1
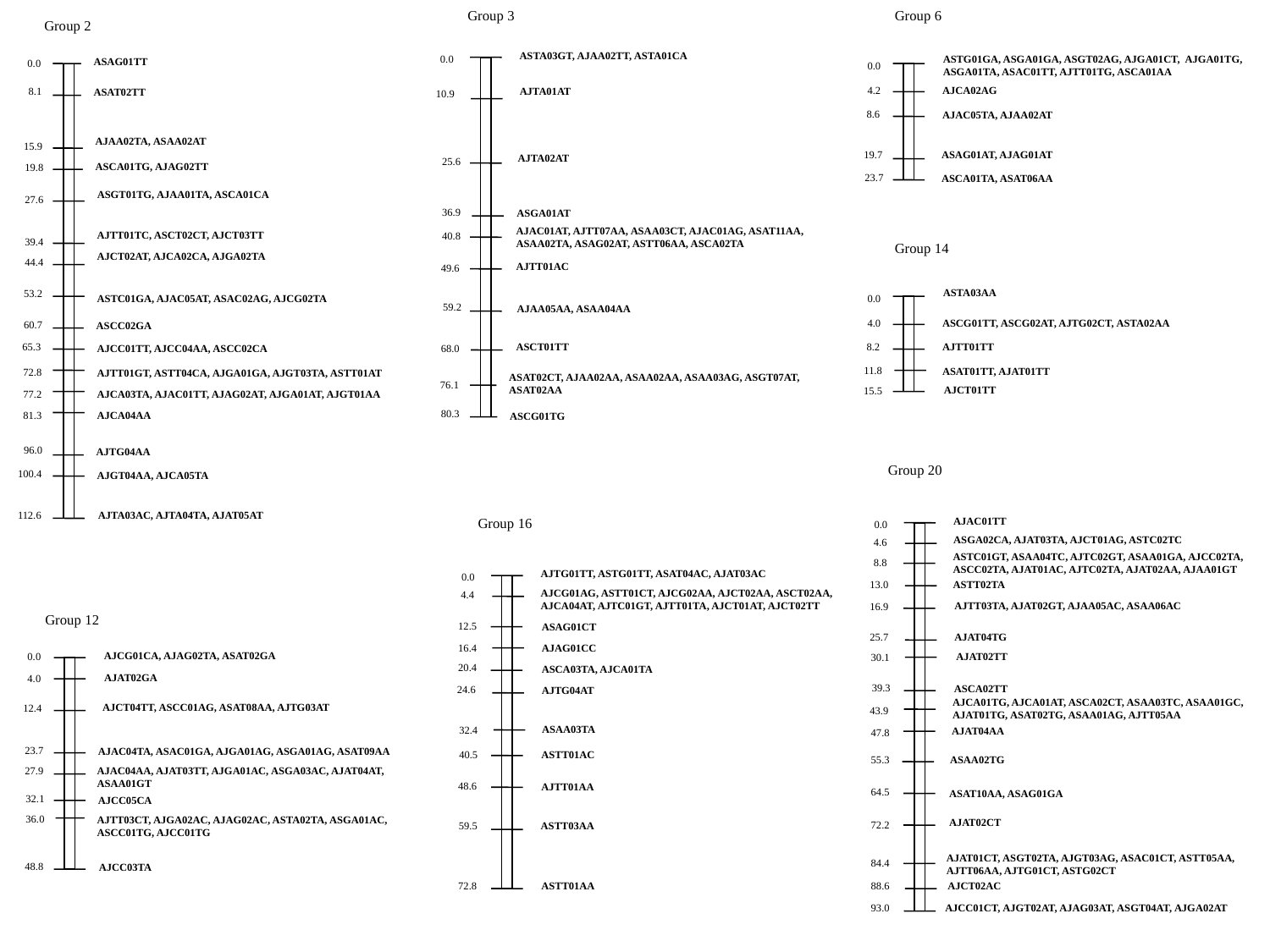

Group 3
Group 6
ASTG01GA, ASGA01GA, ASGT02AG, AJGA01CT, AJGA01TG,
ASGA01TA, ASAC01TT, AJTT01TG, ASCA01AA
0.0
4.2
AJCA02AG
8.6
AJAC05TA, AJAA02AT
19.7
ASAG01AT, AJAG01AT
23.7
ASCA01TA, ASAT06AA
Group 2
ASAG01TT
0.0
8.1
ASAT02TT
AJAA02TA, ASAA02AT
15.9
ASCA01TG, AJAG02TT
19.8
ASGT01TG, AJAA01TA, ASCA01CA
27.6
AJTT01TC, ASCT02CT, AJCT03TT
39.4
AJCT02AT, AJCA02CA, AJGA02TA
44.4
53.2
ASTC01GA, AJAC05AT, ASAC02AG, AJCG02TA
60.7
ASCC02GA
65.3
AJCC01TT, AJCC04AA, ASCC02CA
72.8
AJTT01GT, ASTT04CA, AJGA01GA, AJGT03TA, ASTT01AT
77.2
AJCA03TA, AJAC01TT, AJAG02AT, AJGA01AT, AJGT01AA
81.3
AJCA04AA
96.0
AJTG04AA
100.4
AJGT04AA, AJCA05TA
112.6
AJTA03AC, AJTA04TA, AJAT05AT
ASTA03GT, AJAA02TT, ASTA01CA
0.0
AJTA01AT
10.9
AJTA02AT
25.6
36.9
ASGA01AT
AJAC01AT, AJTT07AA, ASAA03CT, AJAC01AG, ASAT11AA,
ASAA02TA, ASAG02AT, ASTT06AA, ASCA02TA
40.8
AJTT01AC
49.6
59.2
AJAA05AA, ASAA04AA
ASCT01TT
68.0
ASAT02CT, AJAA02AA, ASAA02AA, ASAA03AG, ASGT07AT, ASAT02AA
76.1
80.3
ASCG01TG
Group 14
ASTA03AA
0.0
4.0
ASCG01TT, ASCG02AT, AJTG02CT, ASTA02AA
8.2
AJTT01TT
11.8
ASAT01TT, AJAT01TT
AJCT01TT
15.5
Group 20
AJAC01TT
0.0
ASGA02CA, AJAT03TA, AJCT01AG, ASTC02TC
4.6
ASTC01GT, ASAA04TC, AJTC02GT, ASAA01GA, AJCC02TA, ASCC02TA, AJAT01AC, AJTC02TA, AJAT02AA, AJAA01GT
8.8
ASTT02TA
AJTT03TA, AJAT02GT, AJAA05AC, ASAA06AC
AJAT04TG
AJAT02TT
30.1
39.3
ASCA02TT
AJCA01TG, AJCA01AT, ASCA02CT, ASAA03TC, ASAA01GC, AJAT01TG, ASAT02TG, ASAA01AG, AJTT05AA
AJAT04AA
47.8
ASAA02TG
55.3
64.5
ASAT10AA, ASAG01GA
AJAT02CT
72.2
AJAT01CT, ASGT02TA, AJGT03AG, ASAC01CT, ASTT05AA, AJTT06AA, AJTG01CT, ASTG02CT
84.4
88.6
AJCT02AC
93.0
AJCC01CT, AJGT02AT, AJAG03AT, ASGT04AT, AJGA02AT
Group 16
AJTG01TT, ASTG01TT, ASAT04AC, AJAT03AC
0.0
AJCG01AG, ASTT01CT, AJCG02AA, AJCT02AA, ASCT02AA, AJCA04AT, AJTC01GT, AJTT01TA, AJCT01AT, AJCT02TT
4.4
12.5
ASAG01CT
16.4
AJAG01CC
20.4
ASCA03TA, AJCA01TA
24.6
AJTG04AT
ASAA03TA
32.4
40.5
ASTT01AC
48.6
AJTT01AA
ASTT03AA
59.5
ASTT01AA
72.8
13.0
16.9
Group 12
AJCG01CA, AJAG02TA, ASAT02GA
0.0
AJAT02GA
4.0
AJCT04TT, ASCC01AG, ASAT08AA, AJTG03AT
AJAC04TA, ASAC01GA, AJGA01AG, ASGA01AG, ASAT09AA
AJAC04AA, AJAT03TT, AJGA01AC, ASGA03AC, AJAT04AT, ASAA01GT
AJCC05CA
AJTT03CT, AJGA02AC, AJAG02AC, ASTA02TA, ASGA01AC, ASCC01TG, AJCC01TG
AJCC03TA
25.7
12.4
43.9
23.7
27.9
32.1
36.0
48.8
